# Transmission of Hepatitis E Virus from Rabbits to Cynomolgus Macaques 

**DOI:** 10.3201/eid1904.120827

**Published:** 2013-04

**Authors:** Peng Liu, Qiu-Ning Bu, Ling Wang, Jian Han, Ren-Jie Du, Ya-Xin Lei, Yu-Qing Ouyang, Jie Li, Yong-Hong Zhu, Feng-Min Lu, Hui Zhuang

**Affiliations:** Peking University Health Science Center, Beijing, People’s Republic of China

**Keywords:** rabbit hepatitis E virus, zoonoses, cross-species transmission, *Cynomolgus* macaques, extrahepatic replication, viruses

## Abstract

The recent discovery of hepatitis E virus (HEV) strains in rabbits in the People’s Republic of China and the United States revealed that rabbits are another noteworthy reservoir of HEV. However, whether HEV from rabbits can infect humans is unclear. To study the zoonotic potential for and pathogenesis of rabbit HEV, we infected 2 cynomolgus macaques and 2 rabbits with an HEV strain from rabbits in China. Typical hepatitis developed in both monkeys; they exhibited elevated liver enzymes, viremia, virus shedding in fecal specimens, and seroconversion. Comparison of the complete genome sequence of HEV passed in the macaques with that of the inoculum showed 99.8% nucleotide identity. Rabbit HEV RNA (positive- and negative-stranded) was detectable in various tissues from the experimentally infected rabbits, indicating that extrahepatic replication may be common. Thus, HEV is transmissible from rabbits to cynomolgus macaques, which suggests that rabbits may be a new source of human HEV infection.

Hepatitis E virus (HEV) is the causative agent of acute hepatitis E, which is endemic to many developing countries and occurs sporadically in some industrialized countries. HEV is a small nonenveloped virus with a positive-sense single-stranded RNA genome of ≈7.2 kb; it is currently classified as the sole member of the genus *Hepevirus*, family *Hepeviridae* (*1*). Thus far, at least 4 genotypes, which comprise a single serotype, of HEV have been identified in mammals: genotypes 1 and 2 are restricted to strains that infect humans, and genotypes 3 and 4 are zoonotic (*2*). More recently, a putative fifth HEV genotype was identified in wild boars in Japan (*3*). HEV from chickens, which is phylogenetically distinct from HEV from mammals, is likely to be classified as a new genus within the family *Hepeviridae* (*4*).

The zoonotic nature of HEV was first confirmed in 1997 with the identification of HEV isolates in swine in the United States, which were most closely related to an isolate of HEV from a person in the United States, and this isolate could experimentally infect nonhuman primates (*5*,*6*). Zoonotic transmission of HEV was further substantiated with the demonstration of HEV infection in persons after they ate undercooked infected meat from wild boars and wild deer (*7*,*8*). Antibodies against HEV have been detected in numerous animal species, including dogs, cats, sheep, goats, horses, cattle, bison, and rats; and HEV strains have been genetically identified from domestic and wild pigs, chickens, deer, mongooses, and rabbits (*4*,*9*). The recent discoveries of HEV-like viruses in rats and fish have further broadened understanding of the host range and diversity of HEV (*10*–*12*).

The first strain of rabbit HEV was isolated from Rex Rabbits on 2 rabbit farms in Gansu, People’s Republic of China (*13*). Additional studies indicated that rabbit HEV was prevalent among various breeds of farmed rabbits throughout much of China, and the prevalence of antibodies against HEV was 57.0% in Lanzhou and 54.6% in Beijing (*13*–*15*). Rabbit HEV has also been isolated from rabbits in Virginia, USA, which showed a high prevalence of antibodies against HEV (36%) and HEV RNA (16.5%) (*16*). Phylogenetic analyses revealed that rabbit HEV was most closely related to genotype 3 HEV, which has been confirmed to infect humans. Furthermore, a recent study indicated that rabbit HEV is antigenically related to the other known animal strains of HEV and is experimentally transmissible to swine (*17*). However, to our knowledge, no study had determined the zoonotic potential of rabbit HEV. Therefore, in this study, we endeavored to ascertain whether rabbit HEV can cross species barriers and infect nonhuman primates and to further clarify the pathogenesis and replication of rabbit HEV in its natural host.

## Materials and Methods

### Virus Inocula

The rabbit HEV strain (CHN-BJ-R14) used in this study was originally recovered from the feces of a farmed Rex Rabbit in the suburbs of Beijing in 2011. The fecal sample was diluted in phosphate-buffered saline (PBS) (pH 7.4) containing 1% bovine serum albumin to make a 10% (wt/vol) suspension. The clarified suspension was subsequently filtered through 0.45-μm and 0.22-μm filters. Titers of the rabbit HEV inoculum were determined by a semiquantitative nested reverse transcription PCR (RT-nPCR) (*18*), and the titer was 10^4^ genome equivalents (GE) per milliliter (mL).

### Animals

Two juvenile male cynomolgus monkeys (*Macaca fascicularis*), weighing 2.0–2.5 kg, designated as Cy1 and Cy2, were obtained from the Beijing Xierxing Institute of Biologic Resources (Beijing, China) for the cross-species infection study. For the rabbit infection study, four 7-week-old specific-pathogen free (SPF) New Zealand white rabbits, weighing 750–1,000 g, were obtained from the Department of Laboratory Animal Science of Peking University Health Science Center. Preinoculation serum and feces specimens were collected once a week for 3 weeks, and all animals were tested for alanine aminotransferase (ALT) to establish a baseline, and were confirmed as negative for antibodies against HEV by an ELISA and negative for HEV RNA by RT-nPCR. The animal experiments were approved by the Committee of Laboratory Animal Welfare and Ethics, Peking University Health Science Center. The regulations of the review committee of Laboratory Animal Welfare and Ethics and the protocol for the review on Laboratory Animal Welfare and Ethics, Peking University Health Science Center, were followed.

### Experimental Inoculation of Nonhuman Primates 

To determine whether rabbit HEV strains are transmissible to nonhuman primates, we inoculated intravenously 2 cynomolgus monkeys, housed separately, with 2 mL of the rabbit HEV inoculum. After inoculation, serial serum and fecal samples were collected 2×/week for 16 weeks.

Serum samples were tested for ALT levels and for IgM and IgG against HEV. All samples were also assayed for HEV RNA by RT-nPCR (*15*).

### Experimental Infection of Rabbits 

To clarify the extrahepatic replication sites of HEV, rabbits were experimentally infected with rabbit HEV as described (*19*). In brief, 4 SPF rabbits, which were housed in separate cages, were divided randomly into 2 groups (2 rabbits per group) and inoculated intravenously with either 1 mL of PBS (negative control) or 1 mL of rabbit HEV inoculum. Serum and fecal specimens were collected weekly after inoculation. Serum samples were tested for ALT activity and HEV RNA. Fecal specimens were also assayed for HEV RNA. If serum and fecal specimens became simultaneously positive for HEV RNA, a complete necropsy was performed of each rabbit. Bile and various different types of tissues and organs, including liver, kidney, small intestine, spleen, stomach, heart, brain, bladder, and lung, were collected and stored at −80°C. To prevent cross-contamination during necropsy, we used individually wrapped, sterile disposable materials and new sterile scalpel blades for each sample.

Approximately 100 mg of each tissue and organ was homogenized in 1 mL of sterile PBS (pH 7.4) to make 10% (wt/vol) suspensions and clarified by centrifugation at 4,500 *g* for 10 min at 4°C. Thereafter, 100 µL of the clarified supernatants was used for total viral RNA extraction, and positive-stranded and negative-stranded HEV RNA were detected by RT-nPCR as described below.

### Determination of ALT Levels

All serum samples were tested immediately for ALT levels with a Hitachi Automatic Clinical Analyzer 7180 (Hitachi High-Technologies, Tokyo, Japan), by using chemical reagents purchased from Roche (Basel, Switzerland), according to the manufacturer’s instructions. Biochemical evidence of hepatitis was recorded when the serum ALT level exceeded the baseline ALT level by >2-fold, as defined by a peak ALT value that was equal to or greater than double the prechallenge values (*19*,*20*).

### ELISA to Detect Antibodies against HEV 

The serum specimens collected from monkeys were tested for IgM and IgG against HEV by using an ELISA based on the virus E2 protein (amino acids 394–606 of HEV open reading frame [ORF] 2) (*20*), according to the manufacturer’s instructions (Wantai, Beijing, China). The serum samples collected from rabbits were also examined for antibodies by using the same assay. Signal-to-cutoff values were calculated, and values >1 were considered positive. Preinoculation baseline serum specimens were used as negative controls for each monkey.

### RT-nPCR to Detect Positive-stranded and Negative-stranded HEV RNA

RNA was extracted from 100 μL of serum, bile, tissue suspension, or 10% fecal suspension by using TRIzol reagent (Invitrogen, Burlington, ON, Canada), and purified RNA was resuspended in 11 μL of RNase-free water. To detect positive-stranded HEV RNA, 11 μL of purified RNA was reverse transcribed at 42°C for 60 min with SuperScript II reverse transcription (Invitrogen) and the external reverse primer P4 or S4 in a reaction mixture of 20 μL. Then, nested PCRs were carried out to amplify the partial fragments of ORF1 (129–373 nt) and ORF2 (5,983–6,349 nt) of the HEV genome by using the 2 sets of specific external and internal primer pairs listed in Technical Appendix Table 1). The PCR parameters for both sets of primers and both rounds of PCR were the same, with an initial incubation at 94°C for 5 min, followed by 30 cycles of denaturation at 94°C for 30 s, annealing at 50°C for 30 s, and extension at 72°C for 40 s, with a final incubation at 72°C for 10 min.

Tissues with detectable positive-stranded HEV RNA were then assayed for negative-sense HEV RNA by RT-nPCR with the same 2 sets of universal primers (Technical Appendix Table 1). The extracted RNA was subjected to cDNA synthesis with the external forward primer P1 or S1. Then parental RNAs were degraded by RNaseH, and this was followed by nested PCR. The amplification conditions for negative-stranded HEV RNA detection were essentially the same as those used in the detection of positive-sense HEV RNA.

The PCR protocol used in this study could detect as few as 10 GE copies of HEV plasmid DNA. Negative and positive controls were included in each assay to exclude the possibility of contamination and failure of amplification. A recombinant plasmid containing HEV ORF1 and ORF2 fragments at a concentration of 10^2^ copies per mL and serum or fecal specimens or tissues from naive rabbits were used as positive and negative controls, respectively. Samples showing a band of the expected size on a 1.5% (w/v) agarose gel were considered positive, and the positive products were directly sequenced.

### Amplification of the Full-Length Genome of Rabbit HEV

To compare the complete genome sequence of the HEV passed in the macaques to that of the inoculum, the fecal sample (rHEV-Cy1) of 1 monkey at 3 weeks’ postinoculation (wpi) and the inoculum (CHN-BJ-R14) were sequenced to determine the full-length genome as reported (*21*). Briefly, total RNA was extracted from 120 μL of the rabbit HEV inoculum and a 10% monkey fecal suspension in PBS by using the Total RNA Isolation System (Promega, Madison, WI, USA). cDNA was synthesized from 12 μL of purified RNA by using 1 μL (200 U) of Moloney murine leukemia virus reverse transcription (Promega) and 2 μL (10 pmol/L) of OligodT primer. With 6 sets of specific external and internal primer pairs (Technical Appendix Table 2), a set of nested PCRs were performed by using the first-strand cDNA to amplify the entire viral genome. The nested PCR was done as described (*21*). The nucleotide sequences at the 5′ and 3′ termini of the genome were determined by using a rapid amplification of cDNA ends (RACE) kit (Invitrogen), according to the manufacturer’s instructions.

### Sequence Analyses

The expected PCR products amplified from the inoculum and monkey fecal sample at 3 weeks wpi were purified and ligated into a pGEM-T vector (Promega). At least 3 positive clones for each region of the viral genome were sequenced commercially in both directions by using an automated DNA sequencer (ABI model 3730 sequencer; Applied Biosystems, Foster City, CA, USA).

Nucleotide sequences were assembled and analyzed with the MEGA 4.0 and ALIGNX software (Vector NTI package version 9.0; Invitrogen). ORFs were identified by using the EMBOSS software (version 5.0.0; emboss.sourceforge.net). The full-length genomic sequences of CHN-BJ-R14 and rHEV-Cy1 reported in this study have been deposited in GenBank under accession nos. JX109834 and JX121233, respectively.

## Results

### Cross-Species Transmission of Rabbit HEV to Nonhuman Primates

In both of the macaques inoculated with rabbit HEV, hepatitis developed, as determined on the basis of ALT elevation, viremia, fecal shedding of viruses, and seroconversion (Figure). Dramatic elevations in serum ALT were observed 5 and 10 wpi for both monkeys, with a peak value of 135 U/L at 9 wpi for monkey Cy1 and 97 U/L at 5.5 wpi for monkey Cy2.

**Figure:**
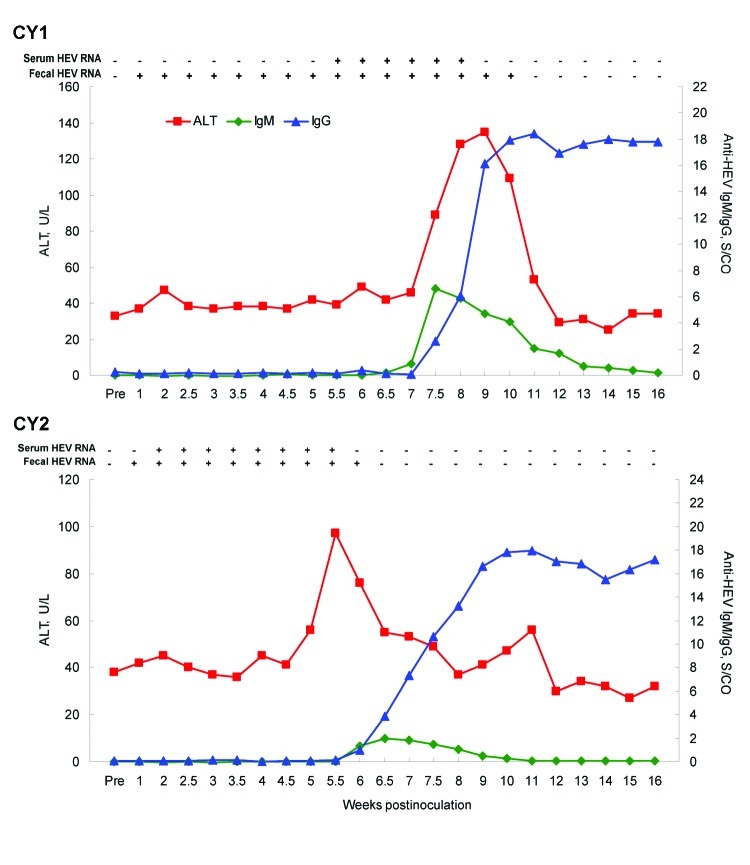
Cross-species transmission of rabbit hepatitis E virus (HEV) to 2 cynomolgus macaques (Cy1 and Cy2). Alanine aminotransferase (ALT) levels are plotted as U/L. The baseline ALT levels were 33 U/L and 38 U/L for Cy1 and Cy2, respectively. The titers of HEV IgM and IgG are plotted as ELISA signal-to-cutoff (S/CO) values. Presence and absence of HEV RNA in serum or feces are indicated by + and – signs, respectively.

Before inoculation, both monkeys were seronegative for HEV and became seropositive for antibodies against HEV at 6–7 wpi. IgM against HEV was detectable from 7 to 12 wpi for Cy1 and from 6 to 8 wpi for Cy2. The rise in IgM against HEV was followed closely by a strong response of IgG against HEV for Cy1, whereas both responses occurred at about the same time for Cy2. The IgG level against HEV remained markedly elevated at the end of the 16-week experiment.

Serum and fecal samples taken before inoculation from both monkeys were negative for HEV RNA. Viremia and fecal shedding of viruses were detected in both monkeys after intravenous inoculation. Fecal excretion of rabbit HEV, indicative of replication, was first detected at 1 wpi and persisted for 5–9 weeks. HEV viremia was first detected at 5.5 wpi for Cy1 and at 2 wpi for Cy2 and lasted for 2.5–3.5 weeks. The partial sequences of the PCR products from both monkeys shared 99%–100% nucleotide identity with the original inoculum.

### Sequence Analyses of Rabbit HEV during Cross-Species Transmission

To analyze mutations in the rabbit HEV genome that appeared during a single passage between the 2 different host species, we sequenced rabbit HEV strains recovered from the inoculum (CHN-BJ-R14) and from experimentally infected cynomolgus monkeys (rHEV-Cy1) over the entire genome. The CHN-BJ-R14 and rHEV-Cy1 isolates had the same genomic length of 7,284 nt, excluding the 3′ poly (A) tail, and contained 3 ORFs—ORF1, ORF2, and ORF3—which encoded proteins of 1,722 aa (nt 26–5194), 660 aa (nt 5232–7214), and 113 aa (nt 5221–5562), respectively. The 5′ untranslated region (UTR) and 3′ UTR comprise 25 nt and 71 nt, respectively. Sequence analyses showed that CHN-BJ-R14 and rHEV-Cy1 shared 99.8% nucleotide identity with each other. Comparison of the complete genome sequence of rabbit HEV passed in the macaques (rHEV-Cy1) with that of the inoculum (CHN-BJ-R14) revealed 18 nt mutations over the entire genome, resulting in 9 nonsynonymous amino acid changes. ORF1 harbored 16 of the 18 nt mutations; 11 were in the helicase domain and in the RNA-dependent RNA polymerase domain (Table).

**Table:** Comparison of the complete genome sequence of rabbit HEV passed in macaques with that of the inoculum*

Nucleotide position†	Genomic region	Nucleotide			Amino acid	
		CHN-BJ-R14‡	rHEV-Cy1§		Position†	Substitution
614	ORF1-MeT	C	T		197	Silent
957	ORF1-Y	T	C		311	Thr to Ile
1667	ORF1-PCP	T	C		548	Silent
1875	ORF1	T	C		617	Pro to Leu
2706	ORF1-X	G	A		894	Asp to Gly
3553	ORF1-Hel	A	T		1176	Silent
3571	ORF1-Hel	C	T		1182	Silent
3859	ORF1-RdRp	C	A		1278	Silent
3889	ORF1-RdRp	C	T		1288	Silent
3972	ORF1-RdRp	G	A		1316	Glu to Gly
4215	ORF1-RdRp	C	T		1397	Leu to Pro
4285	ORF1-RdRp	A	G		1420	Silent
4414	ORF1-RdRp	T	C		1463	Silent
4427	ORF1-RdRp	C	T		1468	Tyr to His
4882	ORF1-RdRp	T	C		1619	Silent
5028	ORF1-RdRp	T	C		1668	Ala to Val
5531	ORF2	C	T		100	Silent
	ORF3				104	Ala to Val
5713	ORF2	T	A		161	Ile to Asn

*HEV, hepatitis E virus; ORF, open reading frame; Thr, Threonine; Ile, Isoleucine; Pro, proline; Leu, leucine; Asp, aspartic acid; Gly, glycine; Glu, glutamic acid; Tyr, tyrosine; His, histidine; Ala, alanine; Val, valine; Asn, asparagine. †Nucleotide or amino acid position according to the rabbit HEV CHN-BJ-R14 strain. ‡CHN-BJ-R14, HEV isolate recovered from the rabbit HEV inoculum in this study. §rHEV-Cy1, HEV isolate recovered from the fecal sample of 1 monkey at 3 wpi in this study. ¶Putative domains in ORF1. MeT, methyltransferase domain; Y, Y domain; PCP, papain-like cysteine protease domain; X, X or macro domain; Hel, helicase domain; RdRp, RNA-dependent RNA polymerase domain.

Nucleotide BLAST (http://blast.ncbi.nlm.nih.gov/Blast.cgi) analysis showed that CHN-BJ-R14 and rHEV-Cy1 were most closely related to genotype 3 HEV with a maximum nucleotide identity of 81%, with the exception of 3 other rabbit HEV strains isolated in Gansu (*13*) and Beijing (*21*). However, several unique features possessed only by rabbit HEVs, but not genotype 3 or other HEV strains, were observed in the 2 rabbit HEV isolates of this study. These features, discovered in a previous study (*21*), were characterized by an insertion of 31 aa in ORF1 (929–959 aa) and a unique A residue at nt 13 (sites based on CHN-BJ-R14) in the 5′ UTR (data not shown).

### Extrahepatic Replication of HEV in Experimentally Infected Rabbits

Both control rabbits remained negative for HEV RNA throughout the study. Viremia and fecal shedding of HEV were detected in rabbits inoculated with the rabbit HEV inoculum. Both rabbits were necropsied, at 5.5 wpi and 12 wpi, respectively, when ALT elevation was observed, and HEV RNA was detected simultaneously in serum and feces. Bile and 9 different types of tissues and organs were collected and tested for positive-stranded HEV RNA. Positive-stranded HEV RNA was detected in bile and in 5 of the tissues—liver, kidney, small intestine, spleen, and stomach. Detection of positive-stranded HEV RNA from various tissues and organs did not indicate that the virus was replicating in these tissues because contamination of the tissue samples by virus circulating in the blood could not be ruled out. To further identify the replicating sites of HEV, we screened for negative-stranded RNA, which is an intermediate product during HEV replication, in all tissues that were positive for the positive-stranded HEV RNA. Negative-stranded RNA was also detectable in the 5 types of tissues. The positive products were sequenced and found to be identical to the original inoculum.

## Discussion

Since the first animal strain of HEV, swine HEV, was identified from a pig in the United States in 1997 (*5*), the increasing identification of HEV infection among a wide range of animals, including pigs, chickens, wild boar, and deer (*4*), has raised public health concern for zoonoses and food safety (*22*,*23*). The recent discovery of rabbit strains of HEV in China (*13*) and the United States (*16*) showed that farmed rabbits are another key reservoir of HEV. In our previous study, phylogenetic analysis of the genome of rabbit HEV suggested the potential for cross-species transmission of rabbit HEV (*21*). A recent study also demonstrated that rabbit HEV can cross species barriers and infect SPF pigs (*17*). In the study described here, we showed that under experimental conditions, rabbit HEV is transmissible to cynomolgus macaques, which can serve as surrogates for humans. This finding suggests that rabbit HEV may be a new source of human HEV infection.

In both cynomolgus monkeys infected in this study with 10^4^ GEs of rabbit HEV, typical acute hepatitis E developed. The patterns of HEV infection in cynomolgus monkeys infected with rabbit HEV were similar to those of animals inoculated with HEV strains of genotypes 1–4, that is, characterized by fecal excretion of virus, followed by viremia and liver enzyme elevation and finally by seroconversion (*24*–*27*). Although the same viral doses were inoculated into both monkeys, the overall course of disease varied somewhat, findings in accord with those of previous studies (*28*). In an earlier study, cross-species infection of pigs infected with rabbit HEV showed a delayed onset and short duration of viremia and fecal virus shedding and an absence of seroconversion (*17*), which differed from findings observed in infected monkeys of this study. The differences might suggest that pigs are less susceptible than nonhuman primates to rabbit HEV. However, because the inocula in both the current study and in other studies (*17*,*19*) have not yet been titrated for infectivity and because HEV infections are virus dose dependent (*18*), additional studies should be performed to determine the infectivity titer of rabbit HEV and to demonstrate whether the rate of inducing hepatitis increases with virus dose of infection.

In the current study, although comparison of the full-length sequences of rHEV-Cy1 and CHN-BJ-R14 showed 99.8% nucleotide identity, 18 nt changes, resulting in 9 nonsynonymous amino acid substitutions, were found in the genome of HEV. These results suggest that adaptation of rabbit HEV to growth in cynomolgus monkeys may be associated with a certain number of mutations. Eleven of the 16 mutations fell within ORF1, accompanied by 4 nonsynonymous substitutions, mapped to the helicase region and the RNA-dependent RNA polymerase region, which are essential for efficient replication of the genomes of HEV (*29*). Moreover, although most mutations are expected to be in the third codon position, of the 16 substitutions in ORF1, 7 occur at the first codon position and 3 at the second codon position. These facts may indicate that positive selection is operating in the infection of the cynomologus monkeys with the rabbit HEV inoculum. A recent study revealed that high-throughput sequencing of isolates from bile and feces from 2 pigs experimentally infected with human HEV of genotype 3f shared the same full-length consensus sequence as in the human sample, although a limited spectrum of mutations were observed during the interspecies transmission (*30*). The genomic sequences in this study were determined by sequencing several randomly selected positive clones, which is much less extensive than high-throughput sequencing; consequently, additional studies will be needed to verify whether the sequence changes that occurred after cross-species transmission of rabbit HEV to cynomolgus monkeys are adaptive mutations or result from the quasispecies structure of HEV.

Previous data from studies performed with pigs infected with human and swine HEV indicated that HEV can replicate in tissues and organs other than the liver (*31*). Recently, extrahepatic manifestations associated with HEV infection, including neurologic disorders (*32*) and acute pancreatitis (*33*), also suggested that HEV could replicate in extrahepatic tissues. The discovery of rabbit HEV opened a new avenue for the study of HEV replication and pathogenesis. Rabbits were used as an animal model to study the extrahepatic replication of HEV in this study. Positive-stranded HEV RNA was detected in the liver, bile, kidney, small intestine, spleen, stomach, serum, and feces from experimentally infected rabbits. Furthermore, negative-stranded HEV RNA, indicative of replication, was also discovered in the same tissues, which provided additional evidence for extrahepatic replication of HEV in its natural host. Considering the extrahepatic replication of HEV found in this study and the other reports of extrahepatic manifestations of HEV infection in humans (*34*), clinicians should consider the possibility of HEV infection in patients with nonhepatic diseases, especially patients with acute pancreatitis, neurologic syndromes, thrombocytopenia, hemolysis, and autoimmune manifestations.

In conclusion, the successful infection of cynomolgus macaques with rabbit HEV suggests that humans might be at risk for infection with rabbit HEV. Further, rabbit HEV was detectable in multiple rabbit tissues and organs, indicating extrahepatic replication may be a common feature of rabbit HEV. These findings raise additional concern for zoonotic transmission of HEV infection among persons who have occupational exposure to rabbits or persons who eat undercooked rabbit meat. Future studies should be conducted to investigate rabbit HEV infection in human populations and assess whether close contact with rabbits is a risk factor for HEV infection.

Technical Appendix Sequences of primers for reverse transcription nested PCR to detect positive-strand and negative-stranded hepatitis E virus RNA and position and nucleotide sequence of primers for nested PCR and rapid amplification of cDNA ends.
