# Supplementary material for: Transmission of Hepatitis E Virus from Rabbits to Cynomolgus Macaques
Source: Emerg Infect Dis. 2013 Apr;19(4):559–65. doi: 10.3201/eid1904.120827 (PMC3647409; doi:10.3201/eid1904.120827)
Supplement: Technical Appendix — Sequences of primers for reverse transcription nested PCR to detect positive-strand and negative-stranded hepatitis E virus RNA and position and nucleotide sequence of primers for nested PCR and rapid amplification of cDNA ends. [file 12-0827-Techapp-s1.pdf]

Article DOI: http://dx.doi.org/10/3201/eid1904.120827

Transmission of Hepatitis E Virus from Rabbits to Cynomolgus Macaques

Technical Appendix

Technical Appendix Table 1. Sequences of primers for RT-nPCR to detect positive-stranded and negative-stranded HEV RNA*

Primer	Sequence	Site (nt)†
P1	5’-AGGCTCCTGGCRTYACTACTG-3’	48-69
P2	5’-GYCYTKGCGAATGCTGTG-3’	104-111
P3	5’-GTRTACCAVCGCTGAACRTC-3’	384-365
P4	5’-CATNGCYTCNGCRACATC-3’	559-542
S1	5’-TTATGCCCAGTARCGYGTTG-3’	5724-5743
S2	5’-GGNTGGCGYTCNGTYGAGAC-3’	5950-5969
S3	5’-CDGCCGACGAAATYAATTCTG-3’	6355-6335
S4	5’-CCCTTRTCRTGCTGAGCGTT-3’	6452-6433

*RT-nPCR, nested reverse transcription PCR; HEV, hepatitis E virus.

†Site based on the genome of China swine strain (GenBank accession no.: AJ272108). R = A/G; Y = C/T; K = G/T; D = G/A/T; V = G/A/C; N = A/C/T/G.

Technical Appendix Table 2. Position and nucleotide sequence of primers for nested PCR and RACE*

Set	Primer position†	Primer sequence (5′→3′)	TM	Product size, bp
1	Set1-S1/48	AGG CTC CTG GSM TCM CAA CCG	65.8	1,191
	Set1-S2/104	GCC CTG GCG AAT GCT GTG	61.9	
	Set1-A1/1301	GGC CRG GGA TRT ART CRC G	61.9	
	Set1-A2/1330	CCG GCA CTG CGC RTA RAA YT	60.9	
2	Set2-S1/1046	ATG ACY TAC CTT CGC GGT ATT AG	59.3	2,431
	Set2-S2/1094	GTC GCY AAC GAG GGY TGG AA	61.9	
	Set2-A1/3524	TRG TGG TCT CWG TGA AWG TRG CSC CCT	66.6	
	Set2-A2/3539	CWG CCG TCG CRA TRA TWG TGG TCT C	65.3	
3	Set3-S1/3090	GGT TTG CTG CRT TTA CRC CCC ACT C	65.3	1,753
	Set3-S2/3137	CGK CGT GTT GTM ATC GAT GAR GC	62.8	
	Set3-A1/4890	GWC CRG CGA ACC GGA CGA CAT	64.9	
	Set3-A2/4996	AAC ACA SAC CTG AGC AAC ATT CGT	60.3	
4	Set4-S1/4625	CCC GGY ACA CTA CTT TGG AAC AC	62.8	1,543
	Set4-S2/4819	YGG GCT CTA TGC CGG AGT YGT GGT	67.1	
	Set4-A1/6361	CCGG CCG ACG AAA TTA ATT CTG	60.1	
	Set4-A2/6458	CCC TTG TCY TGC TGY GCR TT	60.9	
5′RACE	5′race-A1/524	CAC AAG TCA TGT AAA GAG TAG AGA GC	60.4	540
	5′race-A2/564	TGC GCC ATT GCC TCC GCA ACG TC	67.3	
3′RACE	3′race-S1/6211	ACA ACT ACG GCG GCA ACA CGG TTT ATG	65.0	1,000
	3′race-S2/6299	CGT TGT TTA ACT TAG CTG ATA CAC TTC TC	60.6	

*Primers used for amplifying the entire viral genome of hepatitis E virus (HEV); RACE, rapid ampli cation of cDNA ends; TM, melting temperature.

†The nucleotide positions are in accordance with rabbit HEV isolate ch-bj-n1 (GU937805). K = G/T/U, R = A/G, M = A/C, S = C/G, W = A/T, Y = C/T/U.
